# Comparison of the Safety and Effectiveness of Labor Induction With 25 μg Versus 50 μg of Oral Misoprostol in Women With Premature Rupture of Membranes: A Randomized Controlled Trial

**DOI:** 10.1002/hsr2.70817

**Published:** 2025-05-05

**Authors:** Erfane Ebadi, Maryam Rahimi, Sahar Hosseini, Maryam Mazloomi, Niousha Jamshidnezhad, Fatemeh Jayervand

**Affiliations:** ^1^ Shahid AkbarAbadi Clinical Research Development Unit (SHACRDU) School of Medicine, Iran University of Medical Sciences Tehran Iran; ^2^ Firoozabadi Clinical Research Unit (FACRU) School of Medicine, Iran University of Medical Sciences Tehran Iran; ^3^ Department of Gynecology and Obstetrics School of Medicine, Iran University of Medical Sciences Tehran Iran

**Keywords:** childbirth, misoprostol, pregnancy complications

## Abstract

**Background and Aims:**

The recommended dosing regimens for oral misoprostol in labor induction include 25 μg every 2 h and 50 μg every 4 h. However, there is no specific protocol for these regimens at Shahid Akbarabadi Hospital. This study aimed to assess the safety and effectiveness of the two dosing protocols for labor induction in women with premature rupture of membranes (PROM) and establish a tailored protocol to minimize maternal and neonatal complications.

**Methods:**

This randomized controlled trial was conducted at Shahid Akbarabadi Hospital from 2021 to 2023, including pregnant women with term singleton pregnancies and confirmed PROM. Participants were randomly assigned to receive either 25 μg of oral misoprostol every 2 h (up to 12 doses) or 50 μg every 4 h (up to 6 doses). The primary outcome was the mode of delivery. Secondary outcomes included induction‐to‐delivery time, uterine tachysystole, postpartum hemorrhage, and neonatal complications. Statistical analysis was performed using chi‐square and *t*‐tests for comparisons between groups.

**Results:**

A total of 400 women were enrolled, with 200 in each group. The mean induction‐to‐delivery interval was significantly shorter in the 50 μg group (*p* = 0.001). Uterine tachysystole and postpartum hemorrhage due to atonia were more frequent in the 25 μg group (*p* < 0.05). The rates of cesarean and instrumental deliveries did not differ significantly between groups. The mean ± SD age was 27.1 ± 5.27 years in the 25 μg group and 26.8 ± 5.11 years in the 50 μg group (*p* = 0.639).

**Conclusion:**

Both dosing regimens of oral misoprostol were effective for labor induction, but the 50 μg dose was associated with a shorter induction‐to‐delivery time. The findings suggest that adjusting the misoprostol dosage may reduce complications.

**Clinical Trial Registration Code:** IRCT20221123056587N1.

**Abbreviations**
μg: micrograms
PROM: premature rupture of membranes

## 1 Introduction

Premature rupture of membranes (PROM) is characterized by the rupture of fetal membranes before the onset of uterine contractions [1]. Its prevalence is estimated to be between 8% and 10% in all pregnancies, with approximately 60% occurring in term pregnancies [2]. Failure to intervene promptly in cases of PROM can lead to significant maternal and fetal complications. These include chorioamnionitis, endometritis, heightened risk of cesarean delivery, early neonatal sepsis, and the need for neonatal intensive care unit (NICU) admission [3, 4]. It is imperative to promptly initiate the management of PROM to assess risks and develop an appropriate treatment plan, which may include expectant management or labor induction, depending on clinical circumstances. This approach helps mitigate potential adverse outcomes for the mother and the infant. In managing ruptured membranes, there exists a divergence of perspectives between adopting an expectant stance and opting for immediate labor induction [5].

The expectant strategy involves observing for the natural onset of labor, typically manifesting within 24 h postrupture. Conversely, immediate labor induction entails the administration of prostaglandins for cervical ripening or intravenous oxytocin infusion [6]. Evidence‐based guidelines advocate for the prompt initiation of labor induction. Various investigations have been undertaken to evaluate different approaches to immediate labor induction. Despite oxytocin's widespread usage, its association with escalated labor interventions and limited efficacy in cervical preparation diminishes its favorability [6]. Alternatively, misoprostol, an economical and accessible synthetic prostaglandin E1 analog, is preferred for cervical ripening and labor induction. This method offers several advantages, including enhanced drug safety, decreased duration between membrane rupture and delivery, diminished reliance on epidural anesthesia and oxytocin, lowered likelihood of chorioamnionitis through minimized vaginal examinations, decreased rate of cesarean deliveries, and improved Apgar scores for neonates [7, 8]. These benefits were widely recognized among the majority of patients. Despite discrepancies in opinion concerning the optimal dosage and administration regimen of misoprostol, clinical trials have endorsed the use of 25 μg orally every 2 h for up to 12 doses, or 50 μg every 4 h for up to 6 doses [9, 10, 11]. Recognizing the absence of a standardized protocol at the Shahid Akbarabadi Center's birthing facility, we aimed to establish consensus by evaluating the safety and efficacy of these two methods. This initiative seeks to mitigate potential maternal and fetal complications by devising a tailored protocol.

## 2 Methods

This prospective randomized controlled clinical trial was conducted at Shahid Akbarabadi Hospital, involving pregnant women presenting with PROM between the years 2021 and 2023. The sampling protocol of this study was approved by the Ethics Committee of Iran University of Medical Sciences, Tehran, Iran, under the ethical code: IR.IUMS.FMD.REC.1400.206 and registered with the code: IRCT20221123056587N1, as documented in the Iranian Clinical Trial Registration Center.

Inclusion criteria comprised pregnant women with a gestational age of 36 weeks or more, diagnosis confirmed by amniotic fluid analysis or observation of clear amniotic fluid discharge, a score below 4, indicating an unfavorable cervix, singleton, live pregnancy with cephalic presentation, less than 5 previous deliveries, no previous cesarean deliveries, no contraindications, such as a history of misoprostol allergy, bleeding disorders, or concurrent anticoagulant use. Also, women with twin or multiple pregnancies, any fetal presentation other than cephalic, women with history of allergy to misoprostol, bleeding disorders, or current use of anticoagulant medication, conditions like pre‐eclampsia, severe fetal growth restriction, or active genital herpes, and women with five or more previous deliveries were excluded from the study.

**Figure 1:**
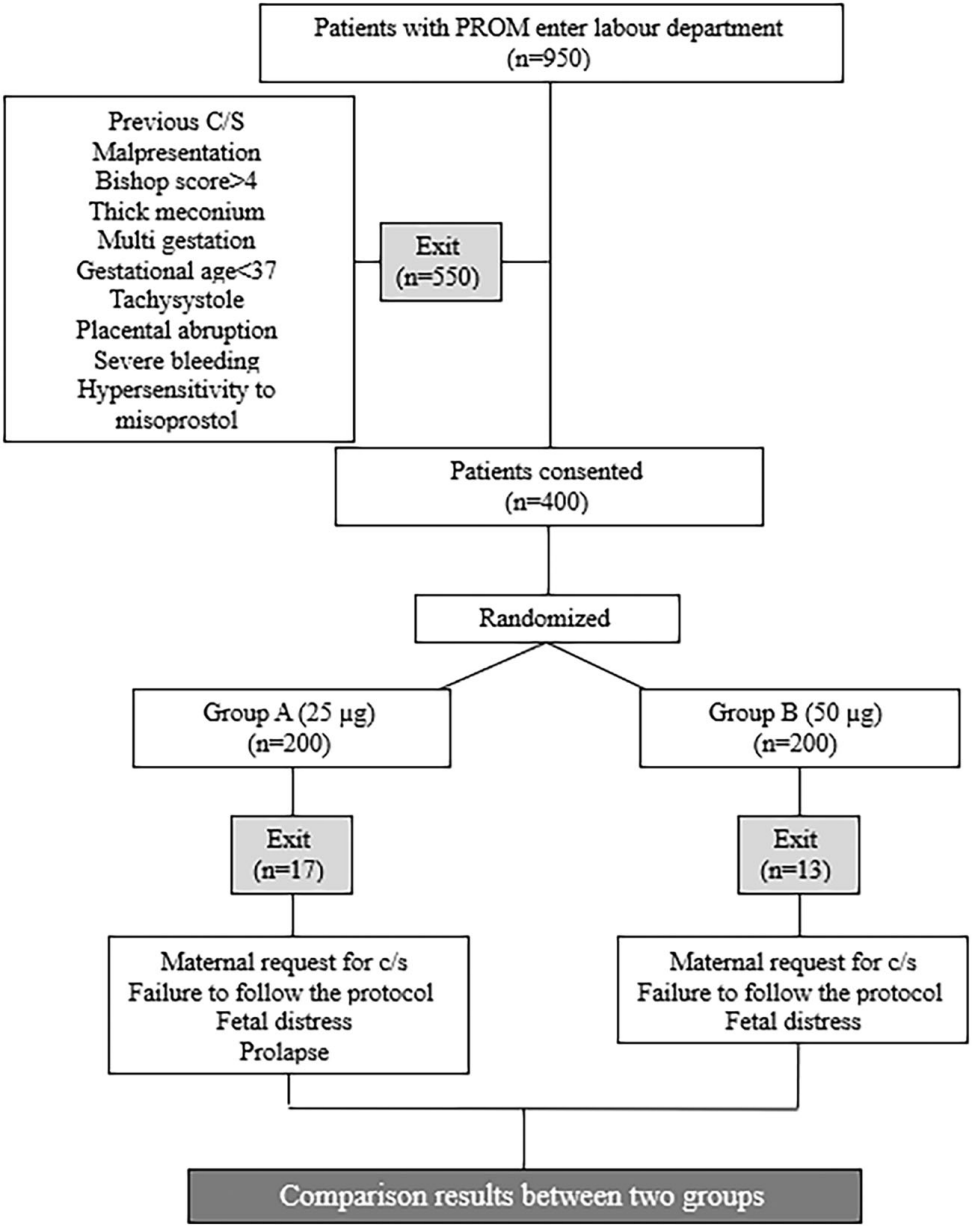
Study design in our study.

A total of 950 patients were initially considered, of which 550 were excluded based on predefined exclusion criteria. The remaining 400 patients were randomly assigned to either the intervention or control group (Figure 1). For the selection of permutations, we first noted their names on six cards and then selected them randomly. The order of removing cards determined the order of choosing intervention groups. The intervention group received oral misoprostol at a dosage of 25 μg every 2 h for a maximum of 12 doses, while the control group received 50 μg every 4 h for up to 6 doses. Dosage administration was supervised by trained midwives. Since both dosing regimens have established efficacy, patients were not at risk of receiving inadequate or no treatment; however, informed consent was obtained from all participants before enrollment.

Baseline data, including demographic and clinical characteristics, were recorded for each patient. The primary outcome measure was the rate of vaginal delivery, while secondary outcomes encompassed rates of instrumental and cesarean deliveries, duration from labor induction to delivery, the incidence of chorioamnionitis (defined as maternal fever, fetal tachycardia, maternal leukocytosis, and histopathological evidence of infection and inflammation in the placenta, fetal membranes, and umbilical cord vessels), uterine tachysystole during labor, neonatal outcomes such as Apgar score, umbilical cord blood gases, NICU admission, and antibiotic usage.

The study was approved by the Ethics Committee of Iran University of Medical Sciences (ethical code: IR.IUMS.FMD.REC.1400.206). Written informed consent was obtained from all participants before enrollment, and the study was registered in the Iranian Clinical Trial Registration Center (IRCT) with the registration number IRCT20221123056587N1. All procedures followed were in accordance with the ethical standards of the Declaration of Helsinki.

## 3 Statistical Analysis

After complete data collection, data analysis was conducted using Statistical Package for the Social Sciences (SPSS) version 22 software. We used independent *t*‐tests for continuous variables (e.g., age, gestational age, Apgar scores), chi‐square tests for categorical variables (e.g., type of delivery, incidence of postpartum hemorrhage), and repeated‐measures ANOVA for subgroup analyses based on parity. We also stated the a priori significance level as *p* < 0.05 and clarified that all tests were two‐sided unless otherwise specified [12].

## 4 Results

The Mean ± SD age of the studied patients in the 25 μg of misoprostol group was 27.1 ± 5.27 years, and in the 50 μg of misoprostol group was 26.8 ± 5.11 years. No statistically significant difference was found between the two groups (*p* = 0.639).

In this study, the mean gestational age of patients in the 25 and 50 μg misoprostol groups was 39.4 ± 1.4 weeks and 38.8 ± 1.45 weeks, respectively, with no significant difference between the two groups (*p* = 0.058).

Results showed that the duration of induction until labor in the 50 μg misoprostol group was shorter than the mg misoprostol group, which was significant (*p* = 0.001) (reduction was observed in both latent and active phases). Also, the incidence of early postpartum hemorrhage in the 50 μg of misoprostol group was higher than in the 25 μg of misoprostol group, which was significant (*p* = 0.004).

Results showed that in the other variables, such as frequency of patient parity, type of delivery, fetal tachycardia, need for oxytocin, fever, leukocytosis, hospitalization in NICU, chorioamnionitis, and uterine tachysystole, there was no significant difference between the two studied groups (*p* > 0.05) (Table 1).

**Table 1:** Pregnancy outcomes in the two studied groups.

Variable	Frequency (%) group		*p* value
	25 μg of misoprostol	50 μg of misoprostol	
Type of delivery			
Cesarean section	54 (29.5)	43 (22.9)	0.365
Normal vaginal delivery	120 (65.5)	132 (70.6)	
Instrumental delivery	9 (4.9)	12 (6.41)	
Parity			
Nulliparous	72 (39.3)	90 (48.1)	0.089
Multiparous	111 (60.6)	97 (51.8)	
Fetal tachycardia			
Yes	33 (18.03)	27 (14.4)	0.348
No	150 (81.9)	160 (85.5)	
Fever			
Yes	35 (19.1)	28 (14.9)	0.288
No	148 (80.8)	159 (85.02)	
Leukocytosis			
Yes	50 (27.3)	39 (20.8)	0.146
No	133 (72.6)	148 (79.1)	
Postpartum hemorrhage			
Yes	14 (7.6)	33 (17.6)	**0.004**
No	169 (92.3)	154 (82.3)	
Need for oxytocin			
Yes	93 (50.8)	(45.9) 86	0.550
No	(49.1) 90	101 (54.01)	
Uterine tachysystole			
Yes	39 (21.3)	69 (36.8)	**0.001**
No	144 (78.6)	118 (63.1)	
Hospitalization in NICU			
Yes	18 (9.8)	17 (9.09)	0.807
No	165 (90.1)	170 (90.9)	
Chorioamnionitis			
Yes	19 (10.3)	14 (7.4)	0.329
No	164 (89.6)	173 (92.5)	

The mean level of 1‐min Apgar score in the 25 μg misoprostol group was slightly higher than the 50 μg misoprostol group, but this difference was insignificant (*p* = 0.146). Also, the mean level of the 5‐min Apgar score in the 50 μg misoprostol group was higher than the 25 μg misoprostol group, but this difference was insignificant (*p* = 0.44). Arterial blood gases between the two studied groups were not significant (*p* = 0.09) (Table 2).

**Table 2:** The mean of Apgar score and arterial blood gases in the two studied groups.

Variable	Mean ± SD group		*p* value
	25 μg of misoprostol	50 μg of misoprostol	
Arterial blood gases	0.57 ± 7.2	0.58 ± 7.23	0.09
1‐min Apgar score	0.7 ± 7.2	1.2 ± 7.4	0.355
5‐min Apgar score	1.2 ± 8.13	1.7 ± 8.22	0.440

To improve results and reduce errors in recalculations, several main variables (delivery type and duration of induction until labor) were examined and reanalyzed based on parity (Table 3).

**Table 3:** The mean of Apgar score and arterial blood gases in the two studied groups.

Variable	Group	Number of patients	Mean ± SD	*p* value
Parity of patients				
Nulliparous	50 μg of misoprostol	72	15.18 ± 3.41	**0.031**
	25 μg of misoprostol	90	13.51 ± 4.71	
	Total	162	14.25 ± 4.25	
Multiparous	50 μg of misoprostol	111	14.06 ± 3.53	
	25 μg of misoprostol	97	12.99 ± 5.14	
	Total	208	13.56 ± 4.38	
Total	50 μg of misoprostol	183	15.50 ± 3.52	
	25 μg of misoprostol	187	13.24 ± 4.93	
	Total	370	13.86 ± 4.33	

**Figure 2:**
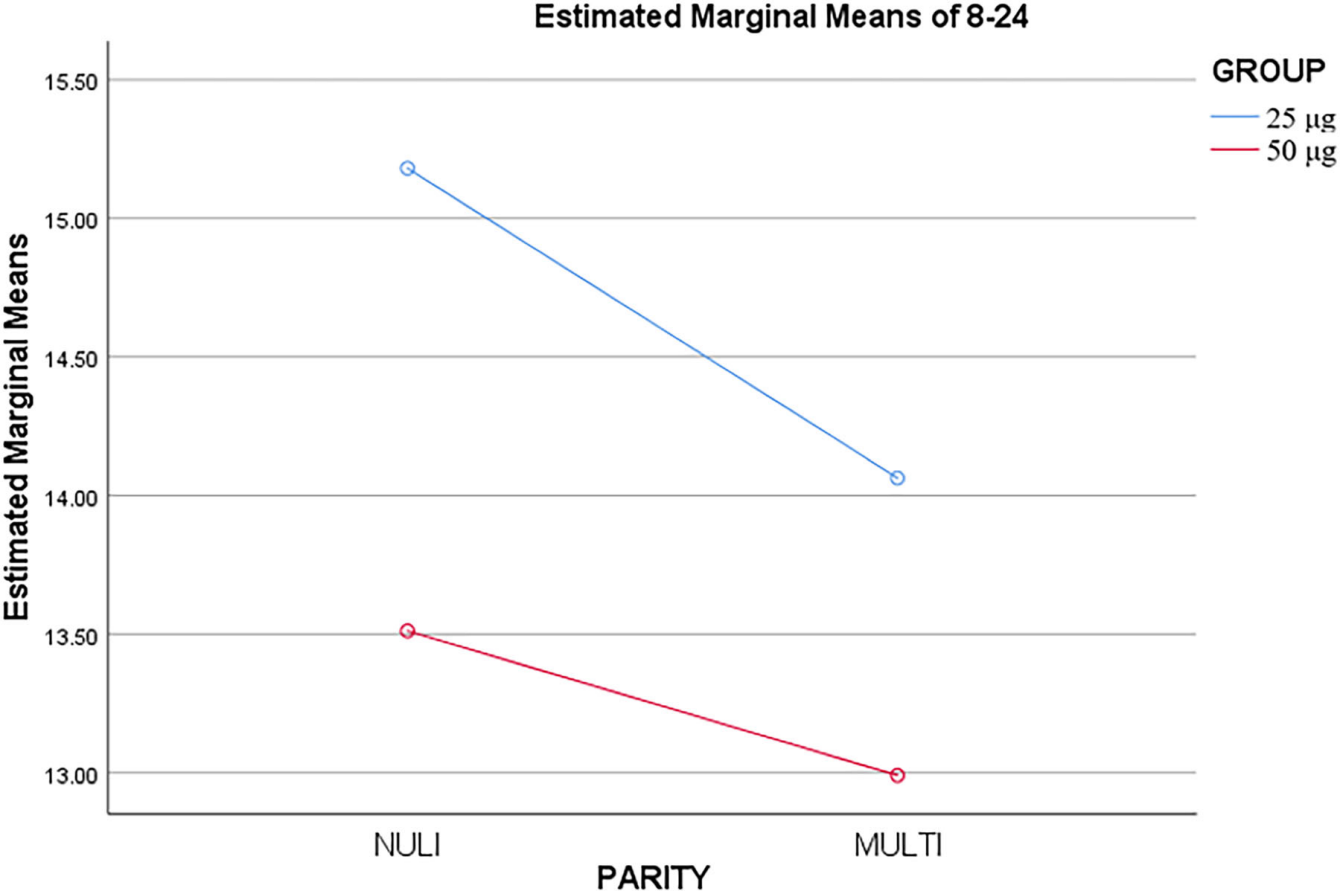
The mean duration of labor induction between two groups based on parity of patients.

The results showed that the duration of labor induction in the multiparous was less than that of the nulliparous, and this mean was lower in the group that received 50 μg of misoprostol than in the group that received 25 μg of misoprostol (*p* = 0.031) (Figure 2).

Instrumental delivery was more frequent in the nulliparous group. Particularly in the 50‐μg group, while the rest of the deliveries were not significantly different between the groups (*p* > 0.05) (Table 4).

**Table 4:** The frequency of delivery type between two groups based on parity of patients.

Variable	Group		Delivery type			Total	*p* value
			Cesarean section	Normal vaginal delivery	Instrumental delivery		
Parity of patients							
Nulliparous	25 μg of misoprostol	Number	26	41	5	72	0.312
		Percentage	36.1	56.9	6.9	100	
		Percentage in total	16.1	25.3	3.1	41.4	
	50 μg of misoprostol	Number	22	58	10	90	
		Percentage	24.4	64.4	11.1	100	
		Percentage in total	13.5	35.8	6.2	55.6	
	Total	Number	48	99	15	162	
		Percentage	100	100	100	100	
		Percentage in total	29.6	61.1	9.3	100	
Multiparous	25 μg of misoprostol	Number	28	81	2	111	0.503
		Percentage	25.2	72.9	1.8	100	
		Percentage in total	13.4	38.9	1	53.4	
	50 μg of misoprostol	Number	21	72	4	97	
		Percentage	21.6	74.2	4.1	100	
		Percentage in total	10.1	34.7	1.9	46.6	
	Total	Number	49	153	6	208	
		Percentage	100	100	100	100	
		Percentage in total	23.6	73.5	2.9	100	
Total	25 μg of misoprostol	Number	54	120	9	183	0.36
		Percentage	29.5	65.5	4.9	100	
		Percentage in total	11.9	32.4	2.4	49.5	
	50 μg of misoprostol	Number	43	132	12	187	
		Percentage	22.9	70.5	6.4	100	
		Percentage in total	11.9	35.6	3.2	50.5	
	Total	Number	97	252	21	370	
		Percentage	100	100	100	100	
		Percentage in total	26.2	68.1	5.7	100	

## 5 Discussion

The present study compared two prescription doses of 25 and 50 μg of oral misoprostol for labor induction in patients with PROM. The results of this study showed uterine tachysystole and the incidence of postpartum hemorrhage in women who received a high dose of misoprostol (50 μg), with a significant difference more than its lower dose. Other studies, including a review by Zhang et al. [13], showed the effectiveness and safety of oral misoprostol in the induction of labor. Also, in another study, Rouzi et al. [14] reported the efficacy and cost‐effectiveness of oral misoprostol in labor induction [13].

According to the results of the present study, it can be concluded that the lower dose of this drug results in fewer side effects. Another important finding was that the duration of induction until delivery was significantly shorter in the group that received a 50 μg dose compared to the 25 μg group This variable was re‐examined separately by parity, yielding a similar result. This difference in the study results can be considered the effectiveness of a higher dose in labor induction.

In Ayaz et al. [15], the delivery time in the group receiving oral misoprostol was less than the vaginal group, so it can be said that in addition to the drug dose, the way the drug is administered may also affect the delivery time. Although a study conducted by Diro et al. [16] found no difference in the success rate of labor induction between the consumption of 25 and 50 μg of misoprostol, the 50 μg dose was associated with shorter first and second stages of labor compared to the other results in the present study. Another result of the present study was the examination of birth complications (fever, leukocytosis, chorioamnionitis, etc.) in the two groups of oral misoprostol, and none of these complications were significantly different in the two groups.

Various prostaglandins, including dinoprostone, are commonly used to manage labor induction. While misoprostol is effective in ripening the cervix and inducing labor, there have been concerns regarding its safety profile, particularly related to the risk of uterine hyperstimulation and adverse neonatal outcomes. Similar concerns have been raised for dinoprostone, which has been widely used for cervical ripening but also carries risks associated with its use.

Recent meta‐analyses have addressed these safety concerns. For instance, Taliento et al. [17] performed a meta‐analysis evaluating the safety and efficacy of different induction agents and highlighted some safety concerns regarding both misoprostol and dinoprostone. Another meta‐analysis by Pergialiotis et al. compared the efficacy and safety of various routes of misoprostol administration. It emphasized the importance of choosing the appropriate method and dosage to minimize risks while achieving effective labor induction [18].

While the type of delivery in the two groups was not significantly different, the number of cesarean deliveries was higher in the 25 μg group, and the prevalence of cesarean delivery in the patients with PROM was about 26.2%. It was caused by the prevalence of non‐progress and failure of induction, fetal distress (persistent category 2, category 3), with a lower prevalence of meconium tic and impending delivery. Also, in Ayaz et al.'s [15] study, there was no difference in the type of delivery with different doses of misoprostol. However, it can be said that the difference in the frequency of delivery type in other studies can be related to the selected statistical population, dosage, and type of drug used. In our studies, the amount of umbilical cord arterial blood gases was measured, which was not measured in any other research, although there was no difference in the mean level between the two study groups.

The previous studies indicate that age plays a significant role in labor induction outcomes. Older women, particularly those aged 35 years or older, tend to have higher rates of induction failure and prolonged time to achieve cervical dilation and delivery. This may be related to age‐associated changes in myometrial contractility or cervical readiness [19]. While our study did not stratify results by age, the lack of significant age differences between the two groups suggests that age‐related disparities did not confound our findings. However, future studies could further explore age as a modifying factor in optimizing misoprostol dosing protocols. However, the findings from the Etrusco et al. [20] study suggest no significant differences in induction outcomes between younger (< 35 years) and older (≥ 35 years) women.

Higher maternal BMI is associated with lower induction success rates, longer induction duration, and increased need for cesarean sections and episiotomies. The mechanisms behind these observations may include altered pharmacokinetics of oral misoprostol in individuals with higher BMI, changes in uterine contractility, or an increased likelihood of labor complications [19]. In our study, while BMI was not analyzed as a covariate, recognizing its impact on induction outcomes highlights the need for personalized dosing strategies based on BMI. Adjusting the dose or frequency of oral misoprostol for women with higher BMI may enhance induction success and reduce adverse outcomes.

One potential limitation of this study is the absence of long‐term follow‐up data beyond the immediate postpartum period. While the immediate outcomes related to labor induction and delivery were assessed, the long‐term effects on maternal and neonatal health, such as the newborns' postpartum complications or developmental outcomes, were not investigated.

## 6 Conclusion

The results indicate that oral misoprostol, at doses of both 25 and 50 μg, is effective for labor induction in patients with PROM, with no significant differences in terms of fetal complications. However, significant differences were found in key maternal outcomes: the 50 μg group experienced a higher incidence of uterine tachysystole and postpartum hemorrhage compared to the 25 μg group. Therefore, the lower dose of 25 μg may offer a safer profile, with fewer maternal side effects, while still achieving effective labor induction. Given the comparable efficacy in labor duration and similar fetal safety outcomes, the 25 μg dose could be considered a more favorable option for managing labor induction in these patients.

## Author Contributions

**Erfane Ebadi:** conceptualization, methodology, data curation, project administration, writing – review and editing, writing – original draft, investigation, supervision. **Maryam Rahimi:** conceptualization, investigation, funding acquisition, writing – original draft, methodology, validation, visualization, writing – review and editing, software, formal analysis, project administration, data curation, resources, supervision. **Sahar Hosseini:** conceptualization, investigation, writing – original draft, writing – review and editing, visualization, methodology, software, resources, supervision, formal analysis. **Maryam Mazloomi:** conceptualization, validation, visualization, writing – original draft, project administration, resources, data curation. **Niousha Jamshidnezhad:** conceptualization, writing – original draft, writing – review and editing, funding acquisition, validation, software, data curation. **Fatemeh Jayervand:** funding acquisition, writing – review and editing, validation, software, resources, supervision, conceptualization, writing – original draft, visualization.

## Ethics Statement

This study was approved by the Ethics Committee of Iran University of Medical Sciences, Tehran, Iran, under the ethical code: IR.IUMS.FMD.REC.1400.206.

## Consent

All authors agree to submit this study article to the journal.

## Conflicts of Interest

The authors declare no conflicts of interest.

## Transparency Statement

The lead author, Maryam Rahimi, affirms that this manuscript is an honest, accurate, and transparent account of the study being reported; that no important aspects of the study have been omitted; and that any discrepancies from the study as planned (and, if relevant, registered) have been explained.

## Data Availability

The data that support the findings of this study are available on request from the corresponding author. The data are not publicly available due to privacy or ethical restrictions.
